# Combined use of ultrasound-assisted washing with in-package atmospheric cold plasma processing as a novel non-thermal hurdle technology for ready-to-eat blueberry disinfection

**DOI:** 10.1016/j.ultsonch.2022.105960

**Published:** 2022-02-25

**Authors:** Jiayi Wang, Zhaoxia Wu

**Affiliations:** a College of Food and Chemical Engineering, Shaoyang University, Shaoyang 422000, China; b College of Food Science, Shenyang Agricultural University, Shenyang 110000, China

**Keywords:** COD, Chemical oxygen demand, CP, Cold plasma, DBD, Dielectric barrier discharge, FC, Free chlorine, AMC, Aerobic mesophilic counts, M&Y, Molds and yeast, MAP, Modified atmosphere packaging, NBT, Nitro blue tetrazolium, PAA, peracetic acid, RNS, Reactive nitrogen species, ROS, Reactive oxygen species, TCD, Total color difference, Ultrasound-assisted washing, Dielectric barrier discharge cold plasma, Disinfection

## Abstract

Ultrasound (US) has limited disinfection efficacy, and it has been recommended to combine it with chemical disinfectants during fresh produce washing. After washing and before packaging, the disinfection effect of US-assisted washing can be weakened; thus, in-package disinfection is important. As a nutritious fruit, there are no packaged blueberries can be directly eaten. Therefore, in this study, blueberry was selected as the model, and the two most commonly used disinfectants (free chlorine [FC] at 10 ppm and peracetic acid [PAA] at 80 ppm) were combined with low-frequency US (25 kHz) during washing, followed by in-package disinfection using dielectric barrier discharge cold plasma (CP). The disinfection efficacy of US-FC and US-PAA against *Escherichia coli* O157:H7 and *Salmonella* Typhimurium was significantly higher than that of US, PAA, or FC alone. The highest disinfection efficacy of CP was observed at the pulse frequency range of 400–800 Hz. For US-FC (1 min) + CP (1 min), an additional 0.86, 0.71, 0.42, and 0.29 log CFU/g of reduction for *E. coli* O157:H7, *S.* Typhimurium, aerobic mesophilic counts, and mold and yeast was achieved, respectively, compared with US-FC (2 min) alone. For US-PAA (1 min) + CP (1 min) an additional 0.71, 0.59, 0.32, and 0.21 log CFU/g of reduction was achieved for the above organisms, respectively, compared with US-PAA (2 min) alone. Quality loss (in total color difference, firmness, and anthocyanin content) was not observed after treatment with US-FC + CP, US-PAA + CP, US-FC, or US-PAA. After treatment with US-FC + CP or US-PAA + CP, the reactive oxygen species (ROS) content was significantly lower than that in the other groups, and antioxidant enzyme activity was significantly higher than that in the other groups, suggesting that in-package CP can activate the blueberry antioxidant system to scavenge ROS, thereby lowering the risk of quality loss. US-CP combination not only improves the disinfection efficacy but also lowers quality loss caused by ROS, without prolonging the processing time.

## Highlights

• Ultrasound washing was used with plasma treatment to disinfect blueberries.
• Combined treatment showed higher microbial count reduction.
• Combined treatment did not negatively affect the quality.
• Combined treatment can activate antioxidant enzymes to scavenge ROS in blueberries.

## 1 Introduction

Foodborne pathogen contamination is the main cause of food safety incidents, approximately 10% of which are caused by the consumption of fresh produce [1]. *Salmonella* is the leading cause of contamination in fresh produce, followed by *Escherichia coli* O157:H7 [2]. According to the recent reports of Center for Disease Control and Prevention (CDC), *Salmonella* caused 1210 illnesses and 250 hospitalizations in 2021, 76.28 and 74.80% of which were caused by the consumption of fresh produce, respectively [3]. *Escherichia coli* O157:H7 caused 63 illnesses and 26 hospitalizations in 2021, 76.28 and 74.80% of which were caused by the consumption of fresh produce, respectively [4]. Therefore, disinfection is an important step before the fresh produce is consumed [5].

Ultrasound (US) is a non-thermal disinfection method, and low frequency (20–100 kHz) US is recommended for the surface disinfection of fresh produce with a treatment time ranging from 1 to 10 min [6], [7], [8], [9]. The disinfection efficacy of US is limited, and the combination of US with other methods is considered for improving the efficacy of US [10]. US is generally used in combination with physical methods, natural compounds, and chemical agents. Recently, Takundwa et al. combined US with the natural compounds nisin and oregano to inactivate *E. coli* on fresh-cut lettuce, observed an improved efficacy compared with a single treatment [11]. Zhang et al [12] found that the natural citral product improved the disinfection efficacy of US against *E. coli* on blueberry. Mild heat was also combined with US to disinfect Rhizopus stolonifera and *E. coli* on sweet potato and sprouting *Brassicaceae* seeds, respectively, and improved efficacy was observed compared with single treatment [13], [14]. Ansorena et al. used citric acid, US, and mild heat to process broccoli and found that the optimum treatment conditions were 7.5 min US, 3 min mild heat, and 1.5% citric acid [15]. During washing, pathogenic bacteria with loose adhesion on the product surface will be circulated with water to contaminate the product, which subsequently enters the washing tank; thus, the disinfection efficacy against the pathogens in the washing water is also critical [16], [17]. Because of the advantages of low-cost and moderate disinfection efficacy, free chlorine (FC) at 10–20 ppm and paracetic acid (PAA) at 80 ppm are effective to prevent cross-contamination and thus were recommended for fresh produce disinfection [17], [18], [19], [20]. US is an effective method for preventing cross-contamination when used in combination with chemical disinfectants. When FC is combined with US, the disinfection efficacy against *E. coli* and *Semonlla* in winter jujube was improved, and the incidence of cross-contamination was completely prevented [5]. Huang et al. [21] found that a combination of US and FC completely prevents cross-contamination of *E. coli* and *Listeria monocytogenes* during lettuce washing.

Fresh produce washing is performed before packaging, and the above-mentioned combination methods are used during the washing process. However, little is known about methods that can be applied to further improve disinfection after washing. Before product sale, the packaging is the last step that can be used to control microbes, and modified atmosphere packaging (MAP) is the most commonly used method. Fan et al. [22] processed fresh-cut cucumbers using US and then packaged them using the MAP method, and found that this combination was effective in controlling microbial growth and reducing quality loss, including weight, ascorbic acid, flavor, firmness, total soluble solids, and total color change. However, the mechanism of MAP is to control microbial growth, not to kill the microbes directly; thus, there is a need for a method to disinfect the packaged fresh produce and determine the combined efficacy of washing and in-package disinfection.

Cold plasma (CP) has been applied as an emerging non-thermal technology to inactivate pathogens in fresh produce. Plasma is an ionized quasi-neutral gas containing positive or negative ions, free electrons, and excited or non-excited molecules [23]. Plasma generation is related to the feeding gas and generation methods. Generation methods include plasma jet and dielectric barrier discharge (DBD) plasma, and the air is the most commonly used feeding gas. The advantage of DBD plasma is that it can disinfect fresh packaged produce. Fresh-cut kiwifruit treated with DBD plasma shows better color retention and a lower darkened area than control kiwifruit [24]. *E. coli* O157:H7 present on packaged lettuce leaves was inactivated by 0.4–0.8 log CFU/g when using DBD plasma [25]. Only recently, Hu et al. [26] used the DBD plasma method to preserve blueberries and found that *Botrytis cinerea* was significantly inhibited, resulting in a decrease in decay from 51 to 11%. However, to our knowledge, no previous study has combined US-assisted washing with in-packaged disinfection using DBD plasma to process fresh produce. Moreover, blueberries are widely consumed worldwide because of their excellent functional properties, including anticancer, antioxidant, and eye protection properties; however, most of the products sold in the market cannot be eaten directly, which does not meet the needs of the consumers. Therefore, in this study, low-frequency US (25 kHz) was combined with two widely used disinfectants (10 ppm of FC and 80 ppm of PAA) to disinfect blueberries during washing, followed by in-package disinfection using CP, and the changes in microbial counts and quality after this sequential treatment were evaluated.

## 2 Materials and methods

### 2.1 Inoculation

Blueberry (Joyvio, Beijing, China) was purchased from a local market on the day of the experiment, and samples with no apparent rotting, wounds, and bruises with a weight of 3.5 ± 0.2 g were selected for the experiment. The sample was rinsed for 30 s under tap water to remove dirt.

*E. coli* O157:H7 (NCTC12900), a non-toxic strain previously used in fresh produce inoculation experiments [27], [28], was selected for this experiment. *Salmonella* Typhimurium (ATCC14028), a quality control strain recommended by the FDA for food safety testing [29], was also selected. One colony strain was inoculated into the nutrient broth (Hopebio, Qingdao, China) and incubated overnight at 37 °C shaking at 150 rpm. After centrifuging at 4000×*g* for 10 min, the obtained cell pellet was washed with sterilized 0.85% NaCl three times and resuspended in sterilized distilled water to adjust the counts to ∼ 10^9^ CFU/mL. Ten blueberries and 150 mL of inoculation solution were placed in a sterilized stomacher bag and manually massaged for 15 min. The inoculated samples were placed in a biosafety cabinet and air-dried for 3 h. The sample was placed under 4 °C for 12 h for bacterial adhesion, and an inoculated sample with 5.8 and 5.7 log CFU/g of *E. coli* O157:H7 and *S.* Typhimurium were obtained, respectively.

### 2.2 Disinfection

#### 2.2.1 Washing water preparation

Water is circulated for use during washing, leading to an increase in chemical oxygen demand (COD); thus, using slurry produce to prepare washing water with a certain COD value is recommended [16], [17], [19], [30]. The blueberry samples were transferred to an analytical mill (A11 basic; IKA, Germany) for processing for 30 s. The obtained homogenate was filtered under vacuum and the slurry was stored at −20 °C until use. It was verified that 10 ppm of FC and 80 ppm of PAA were effective for controlling cross-contamination and with good disinfection efficacy; thus, this was the recommended concentration in the industry [31]. Since FC was consumed during washing, sodium hypochlorite (Sinopharm, Beijing, China) was added to the washing water to adjust the FC concentration to 13.7 ± 1.4 ppm before washing. The COD and PAA concentrations were adjusted to 854 ± 71 and 80 mg/L, respectively. The concentrations of FC, PAA, and COD were determined using *N,N*-diethyl-p-phenylenediamine test kit (Lohand, Hangzhou, China), strips (HKM, Guangzhou, China), and COD test kit (Lohand), respectively.

#### 2.2.2 US-FC and US-PAA processing

Generally, the washing time does not exceed 2 min in practical applications [16]. In this study, 2 min was selected as the washing time based on previous studies [32]. Low-frequency (20–100 kHz) US was more effective for surface disinfection than the high-frequency US [7]; thus, 25 kHz was selected. For US power, softening was observed in the pre-experiment as the power exceeded 400 W; thus, 400 W was selected.

In the pre-experiment, we found that the highest disinfection efficacy was achieved when the sample was dissolved in the disinfection solution at a ratio of 1:20 (w/v) and the disinfection efficacy did not improve further with increased ratio. The sample had to be placed in a cage to prevent it from floating in the ultrasound washer, which led to incomplete contact with the disinfection solution; consequently, we determined that 10 L of water was the minimum volume required to cover the cage. However, 500 g sample must be dissolved in 10 L water to maintain the 1:20 ratio, which is wasteful considering that only small amounts of the sample are needed in microbial and quality analysis. Therefore, 30 blueberries weighing approximately 100 g in total were placed as the sample in a cage (18 × 15 × 5 cm), and placed in an ultrasonic washer (SB-800DTS; Scientiz, Ningbo, China) containing 10 L of washing water. After processing, the samples were rinsed with tap water for 30 s, to remove the sanitizer residue. Then, the sample was dewatered using a sterilized (75% ethanol) manual salad spinner.

#### 2.2.3 US-FC + CP and US-PAA + CP processing

The washing step is a must to remove surface dirt and exert disinfection effects, and the processing time is generally not less than 1 min. If the combined processing time of US-FC + CP and US-PAA + CP exceeded the overall processing time (i.e., 2 min) of US-FC and US-PAA, the production efficiency would be reduced. Thus, the processing times of the first stage (US-FC and US-PAA) and the second stage (CP) were 1 min. US-FC and US-PAA processing were performed as described in Section 2.2.2.

**Fig. 1:**
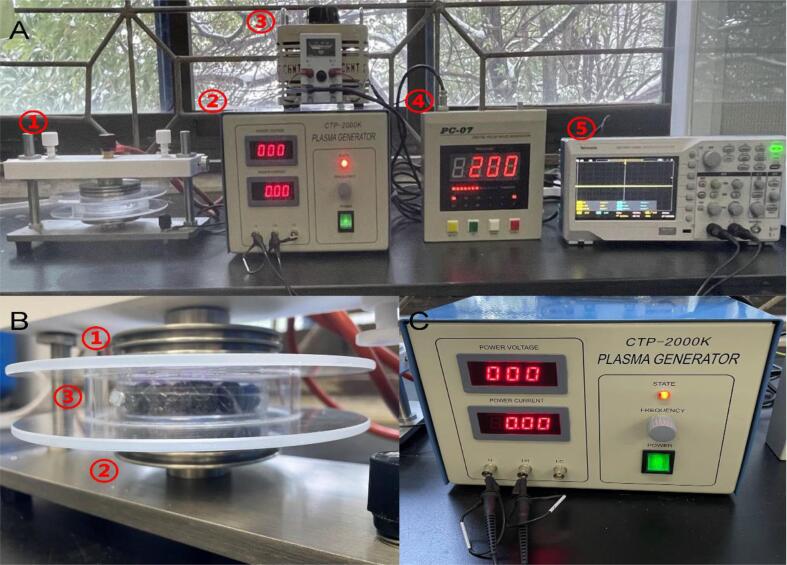
Schematic diagram of the CP equipment. (A) The schematic diagram of each component in the system. 1 is the DBD reactor; 2 is the plasma generator; 3 is voltage booster; 4 is digital pulse wave generator; 5 is the digital oscilloscope. (B) DBD reaction system. 1 is a high voltage electrode; 2 is a low voltage electrode; 3 is a quartz reaction chamber. (C) The plasma generator.

**Fig. 2:**
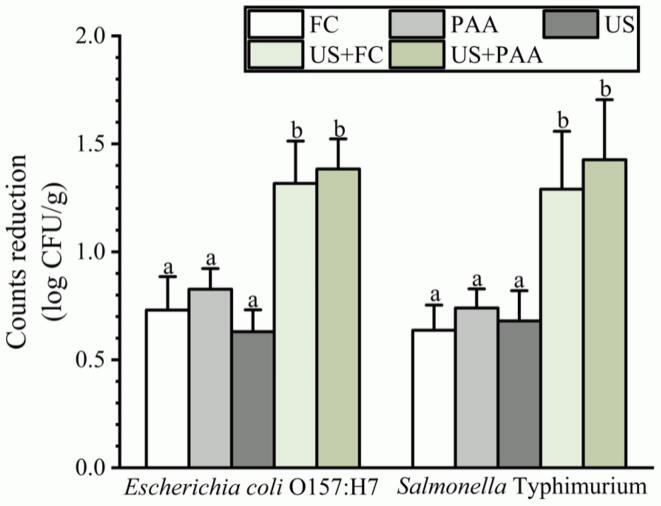
Effects of single and combination treatments against *E. coli* O157:H7 and *S.* Typhimurium on blueberries. Count reduction indicates the difference in microbial counts between the control and treatment groups. The different lowercase letters within the same group indicate significant differences (*P* < 0.05). US, Ultrasound; PAA, peracetic acid; FC, free chlorine.

**Fig. 3:**
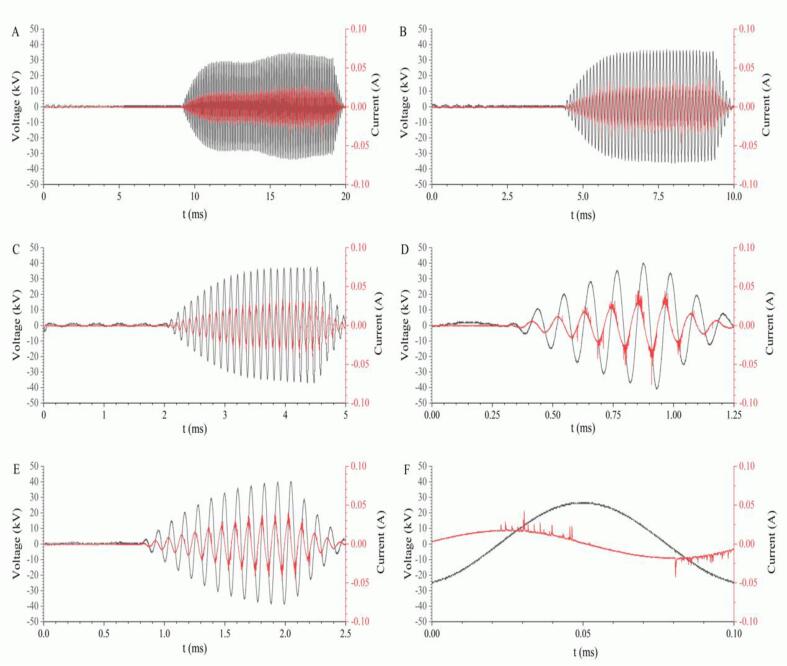
Output voltage and current under different pulse frequency cold plasma treatments. A, B, C, D, E, and F is the output current and voltage under pulse frequencies of 50 Hz, 100 Hz, 200 Hz, 400 Hz, 800 Hz, and 10 kHz, respectively.

**Fig. 4:**
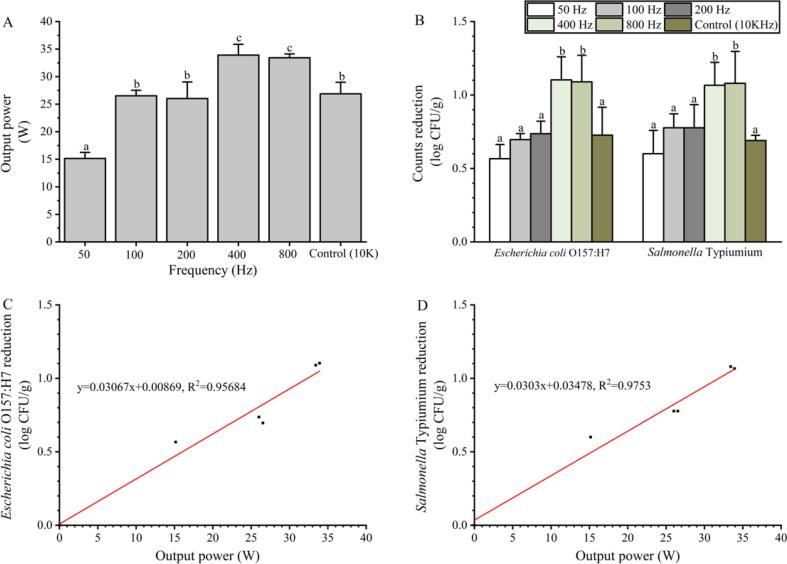
Effects of different pulse frequency plasma treatments against foodborne pathogens on blueberries. (A) The output power corresponding to different pulse frequencies. (B) The microbial reduction after treatment with different pulse frequency plasma. (C and D) The regression curve of the output power and count reduction of *E. coli* and *S.* Typhimurium, respectively. Count reduction indicates the difference in microbial counts between the control and treatment groups. The different lowercase letters within the same group indicate significant differences (*P* < 0.05).

**Fig. 5:**
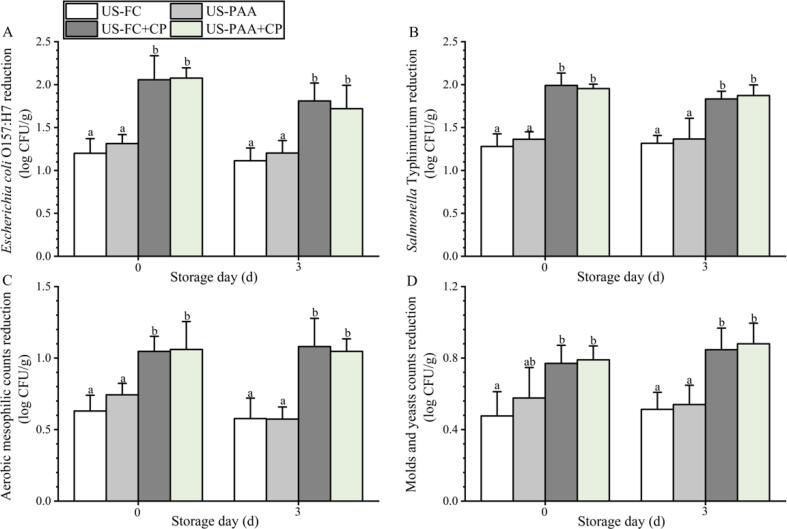
Effects of different combinations on foodborne pathogens and naturally present microbes on blueberries. (A) *E. coli* O157:H7. (B) *S.* Typhimurium. (C) Aerobic mesophilic counts. (D) Molds and yeasts. Count reduction indicates the difference in microbial counts between the control and treatment groups at the same time points. The different lowercase letters within the same group indicate significant differences (*P* < 0.05). US, Ultrasound; PAA, peracetic acid; FC, free chlorine; CP, cold plasma.

A DBD cold plasma system (CTP-2000 KP; Suman, Nanjing, China) was used for plasma treatment. As shown in Fig. 1, this system consisted of a DBD reactor (Fig. 1A-1), plasma generator (10 kHz; Fig. 1A-2), voltage booster (Fig. 1A-3), and digital pulse wave generator (1–999 Hz; Fig. 1A-4). The DBD reactor consisted of high- (Fig. 1B-1) and low-voltage (Fig. 1B-2) electrodes with a diameter of 10 cm. A quartz reaction chamber with a diameter of 18.6 cm was placed between the two electrodes. The plasma generator consisted of two displays (Fig. 1C, showing the input voltage and input current) and an adjustment knob. A digital pulse wave generator was connected to a plasma generator to regulate the pulse frequency (discharge frequency) and duty cycle (discharge gap). A digital oscilloscope (Fig. 1A-5; TBS 1000C, Tektronix, USA) was connected to the plasma generator to obtain the output current and voltage. Before the experiment, as per the manufacturer's instructions, the pulse frequency and duty cycle were set as 200 Hz and 50%, respectively. Then, the knob was first rotated counterclockwise on the plasma generator to find the maximum input current, and then clockwise to establish the input current value as 90% of the maximum input current to generate a stable plasma; the input power at this time was 50 ± 2 W, which was calculated using the following formula:

$$P_{\mathrm{input}}=U_{\mathrm{input}}\times I_{\mathrm{input}}$$

It should be noted that the knob on the plasma generator was not rotated in subsequent experiments to avoid unstable discharge.

To avoid electrical breakdown of the packaging and produce quality loss, the duty cycle was fixed at 50%, according to the manufacturer's guidelines. The pulse frequency was set as 50 Hz, 100 Hz, 200 Hz, 400 Hz, or 10 kHz. Ten blueberries were placed in sterile plastic petri dishes (made of polystyrene, with a diameter of 90 mm and a height of 15 mm) and then wrapped in a single layer using a polyvinyl chloride cling film [33]. As shown in Fig. 1B-3, the packaged sample was packed into a quartz chamber (the distance between the top of the package and the high-voltage discharge site was 0.5 cm). When the plasma pulse frequency was set to different values, the input voltage was adjusted by adjusting the knob on the voltage booster to stabilize the input power to 50 W. After treatment, the samples were stored at 4 °C until analysis. The output voltage and current were visualized using Origin v9.0, and the area was integrated into the software to obtain the output power.

### 2.3 Microbiological analysis

Ten blueberries and 0.85% sterile NaCl solution at a ratio of 1:9 (w/v) were added to a stomacher bag and homogenized for 2 mins under 250 rpm. The bacterial suspensions were then serially diluted. The diluted suspension was surface-plated on modified sorbitol MacConkey agar (Hopebio) and xylose lysine deoxycholate agar (Hopebio) and incubated for 24 h at 37 °C to analyze *E. coli* O157:H7 and *S.* Typhimurium, respectively. For naturally present microbes, 1 mL of suspension was pour-plated on plate count agar (Hopebio) and incubated 48 h at 37 °C to analyze aerobic mesophilic counts (AMC). In addition, 1 mL suspension was pour-plated on Rose Bengal agar (Hopebio) and incubated for 5 days at 28 °C to quantify the amount of molds and yeast (M&Y).

### 2.4 Quality analysis

#### 2.4.1 Liquid nitrogen grinding

Five blueberries were immersed in liquid nitrogen for 30 s and then transferred to an IKA analytical mill for processing (30 s). The ground powder was used for analysis, as described in 2.4.3, 2.4.4.

#### 2.4.2 Firmness and total color change analysis

The L*, a *, and b* color values of the five blueberries were analyzed using a colorimeter (CR400; Konica Minolta, Osaka, Japan). Each sample was analyzed four times for a total of 20 readings per replicate. Total color difference (TCD) was calculated using the following formula:

$$TCD=\sqrt{{\left(L_{1}^{*}-L_{0}^{*}\right)}^{2}+{\left(a_{1}^{*}-a_{0}^{*}\right)}^{2}+{\left(b_{1}^{*}-b_{0}^{*}\right)}^{2}}$$

where $L_{0}^{*}$, $a_{0}^{*}$, and $b_{0}^{*}$ denote color values corresponding to a sample without any treatment and $L_{1}^{*}$, $a_{1}^{*}$, and $b_{1}^{*}$ denote color values corresponding to the treated sample. After TCD analysis, the sample was penetrated 10 mm using a penetrometer (GY-4; Aidebao, Yueqing, China) equipped with a cylindrical probe (3.5 mm diameter) to analyze firmness.

#### 2.4.3 Anthocyanin, hydrogen peroxide, and ${\text{O}}_{2}^{-}$ production rate analysis

Grounded blueberry powder (0.5 g) and 4 mL of 80% methanol were mixed in a pre-cooled tube. After standing for 20 min, the sample was centrifuged at 11000×*g* for 10 min at 4 °C. Exactly 0.5 mL of supernatant was added to 4.5 mL of potassium chloride buffer (0.025 M, pH = 1.0) and sodium acetate buffer (0.4 M, pH = 4.5), and then incubated for 20 min in the dark. The absorbance was measured at 520 and 700 nm. Anthocyanin content was calculated according to the following formula, and the results were defined as mg cyanidin-3-glucoside equivalents per liter:

$$Total\;antocyanin\;content\left(mg/L\right)=\frac{\left[{\left(A_{520nm}-A_{700nm}\right)}_{pH1.0}-{\left(A_{520nm}-A_{700nm}\right)}_{pH4.5}\right]\times M_{w}\times DF\times 1000}{\varepsilon \times L}$$

where Mw is the molecular weight of cyanidin-3-glucoside (449.2), DF is the dilution factor (10 in this study), ε is the molar absorptivity (26900), and L is the cell path length (1 cm in this study).

For hydrogen peroxide and ${\text{O}}_{2}^{-}$ analyses, the methods reported by Zhou et al. [34] were used, with some modifications. For ${\text{O}}_{2}^{-}$ analysis, 0.5 g of grounded powder was mixed with 2.5 mL of 50 mM sodium phosphate buffer (pH 7.8) and then centrifuges at 11000×*g* for 10 min at 4 °C. Next, 1 mL of the supernatant was mixed with 1 mL of 50 mM sodium phosphate buffer and 1 mL of 10 mM hydroxylammonium chloride. After incubation for 20 mins at 25 °C, 1 mL of the above mixture was mixed with 1 mL of 17 mM 4-aminobenzene sulfonic acid and 1 mL of 7 mM -naphthylamine; the absorbance was measured at 530 nm.

For hydrogen peroxide analysis, 0.5 g of grounded powder was mixed with 2.5 mL of pre-cooled acetone and centrifuged at 11000×*g* for 10 min at 4 °C. Then, 1 mL of supernatant was mixed with 0.1 mL of 5% Ti(SO_4_)_2_ and 0.2 mL of concentrated NH_4_OH solution. After reacting for 5 min, the sample was centrifuged at 11000×*g* for 10 min at 4 °C. After discarding the supernatant, acetone was used to wash the precipitate until the solution became colorless. Then, 3 mL of 2 M sulfuric acid was added to dissolve the colorless precipitate and the absorbance was measured at 412 nm.

#### 2.4.4 Catalase (CAT), superoxide dismutase (SOD), and peroxidase (POD) activity analysis

CAT and SOD were analyzed as described by Zhou et al. [34], with some modifications. For CAT analysis, 0.5 g of grounded powder was mixed with 2.5 mL of 0.1 M sodium phosphate buffer (pH 7.8) containing 5 mM DTT and 5% PVP, and then centrifuged at 11000×*g* for 10 min at 4 °C. Then, 0.1 mL of supernatant was mixed with 2.9 mL of 20 mM hydrogen peroxide solution (prepared using 50 mM sodium phosphate buffer [pH 7.8]), and the absorbance was measured at 240 nm within 3 min. The unit was expressed as U/g of fresh weight. One unit of CAT activity was defined as the amount of enzyme that caused a 0.01/min change in absorbance.

For SOD analysis, 0.5 g of grounded powder was mixed with 2.5 mL of 50 mM sodium phosphate buffer (pH 7.8) containing 0.1% (w/v) polyvinyl pyrrolidone, and then centrifuged at 11000 × g for 20 min at 4 °C. The supernatant (0.5 mL) was mixed with 3 mL of reaction solution (130 mM methionine, 100 μM EDTA, 750 μM nitro blue tetrazolium [NBT], and 20 μM riboflavin in 50 mM sodium phosphate buffer [pH 7.8]), and then incubated for 10 min under 4000 lx using a flashlight (Smiling shark, Guangzhou, China). Absorbance was recorded at 560 nm. The unit was expressed as U/g of fresh weight; one U was defined as the enzyme concentration that caused 50% inhibition of NBT reduction.

For POD activity analysis, 0.5 g of grounded powder was mixed with 2.5 mL of acetic acid-sodium acetate buffer (pH 5.5) containing 1 mmol PEG 6000, 4% PVP, and 1% Triton X-100. After centrifuging at 11000×*g* for 10 min at 4 °C, 0.5 mL of the sample was mixed with 3 mL of 25 mM guaiacol, and the absorbance was determined at 470 nm within 5 min. The units were expressed as U/g of fresh weight, and one unit was defined as the amount of enzyme that caused a 0.01/min change in absorbance.

### 2.5 Statistical analysis

Differences between the means of groups were evaluated using one-way analysis of variance using SPSS v.20, and differences in the mean values were analyzed via post hoc Duncan's multiple range test. Statistical significance was set at p < 0.05. All experiments were independently replicated three times. Samples washed with tap water were used as controls.

## 3 Results

### 3.1 US inactivation of pathogens on blueberries in combination with PAA and FC

Tap water washing was the control group and the counts of *E. coli* O157:H7 and *S.* Typhimurium were 5.23 and 5.26 log CFU/g, respectively. As shown in Fig. 2, *E. coli* O157:H7 counts were reduced by 0.73, 0.83, and 0.63 log CFU/g and *S.* Typhimurium counts were reduced by 0.64, 0.74, and 0.68 log CFU/g, after treatment with FC, PAA, or US, respectively. A non-significant difference was observed between the three treatments. After combining US with FC, *E. coli* O157:H7 and *S.* Typhimurium counts were reduced by 1.32 and 1.29 log CFU/g, respectively, which were superior to those obtained via US or FC alone. Similarly, US + PAA led to a more significant microbial reduction (1.38 and 1.43 log CFU/g for *E. coli* O157:H7 and *S.* Typhimurium, respectively) than US and PAA. The microbial reduction between these two combinations was not significantly different.

### 3.2 Inactivation of pathogens on blueberries under different pulse frequency plasma treatments

The waveform graphs for different pulse frequencies are shown in Fig. 3. When the input power and duty cycle were fixed at 50 W and 50%, respectively, the output voltage and current were associated with the changes in pulse frequency. In detail, the maximum peak to peak voltage and current were 68.55 kV and 0.07 A for 50 Hz (Fig. 3A), 73.29 kV and 0.09 A for 100 Hz (Fig. 3B), 74.86 kV and 0.08 A for 200 Hz (Fig. 3C), 78.80 kV and 0.09 A for 400 Hz (Fig. 3D), 81.17 kV and 0.12 A for 800 Hz (Fig. 3E), and 52.01 kV and 0.09 A for 10 kHz, respectively (Fig. 3F).

The group treated without a pulse generator (Fig. 1A-5) was selected as the control (i.e., 10 kHz group). As shown in Fig. 4A, the lowest output power (15.16 W) was observed at a frequency of 50 Hz, and the output power was significantly improved to 26.53 W as the frequency increased to 100 Hz. However, the output power was not further improved as the frequency increased to 200 Hz. Similarly, the highest power (33.91 W) was observed when the power reached 400 Hz, and the power did not improve further as the frequency increased to 800 Hz. Although the power did not increase with the increasing frequency within certain frequency ranges, it showed an overall increasing trend in the range of 0–800 Hz. However, the power decreased to 26.88 W as the frequency increased to 10 kHz, which is similar to the power observed at 100–200 Hz. The count reductions of *E. coli* O157:H7 and *S.* Typhimurium were not significantly improved as the frequency increased from 50 to 200 Hz. As the frequency ranged from 400 to 800 Hz, the count reduction of *E. coli* was 1.09–1.10 log CFU/g, and that of *S.* Typhimurium was 1.07–1.08 log CFU/g, which was significantly higher than the results observed at 50–200 Hz. A moderate microbial reduction (0.73 log CFU/g for *E. coli* O157:H7 and 0.69 log CFU/g for *S.* Typhimurium) was observed as the frequency dramatically increased to 10 kHz, which was similar to that obtained at 50–200 Hz. Since 10 kHz did not lead to an additional microbial reduction, the regression curve was analyzed in the range of 50–800 Hz, and the results indicated that microbial reduction was positively correlated with CP output power (Fig. 4C and D).

### 3.3 US-FC and US-PAA inactivation in combination with CP

The microbial reduction obtained with US-FC treatment was similar to that obtained with US-PAA; thus, these two treatments were combined with CP for subsequent experiments. The microbial reduction was not further improved when the frequency exceeded 400 Hz; thus, 400 Hz was selected in combination with US-FC and US-PAA. On day 0, the counts of *E. coli* O157:H7, *S.* Typhimurium, AMC, and M&Y were 5.09, 5.08, 4.53, and 3.76 log CFU/g, and on day 3, they were 5.30, 5.22, 4.75, and 3.92 log CFU/g, respectively. As shown in Fig. 5, the disinfection efficacy between US-FC and US-PAA was not significantly different, from day 0–3, which was consistent with the results observed in Fig. 2. After combining with CP, the disinfection efficacy was significantly improved on day 0; US-FC + CP led to a 2.06, 1.99, 1.05, and 0.77 log CFU/g reduction for *E. coli* O157:H7, *S.* Typhimurium, AMC, and M&Y, respectively, which was similar to the count reduction caused by US-PAA + CP. After storage for 3 days, the microbial reduction caused by US-FC + CP and US-PAA + CP treatments was significantly higher than that caused by US-FC and US-PAA treatments, and no significant difference was observed between these two combinations.

### 3.4 Blueberry quality change after treatment with different combinations

**Fig. 6:**
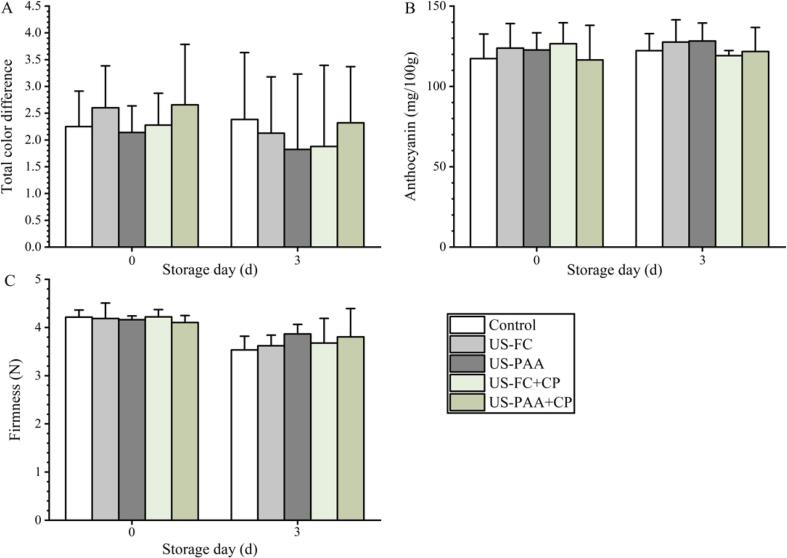
Effects of different combinations on the quality of blueberries. (A) Total color difference. (B) Anthocyanin content. (C) Firmness. No significant differences were observed between the groups (*P* > 0.05) on the same day. US, Ultrasound; PAA, peracetic acid; FC, free chlorine; CP, cold plasma.

As shown in Fig. 6, the TCD values were 2.60 and 2.4 in US-FC and US-PAA groups, which were not significantly different from the control group. After combination treatment with CP, the TCD value did not increase further. On day 3, the TCD values of the US-FC, US-PAA, US-FC + CP, and US-PAA + CP groups were similar to those of the control groups, and no significant differences were observed. Anthocyanin contents in the control group were 117.31 and 122.21 mg/100 g on days 0 and 3, respectively. After washing with US-PAA and US-FC, the content did not change, and until after 3 days the anthocyanin content between these two groups and the control was not significantly different. Similarly, after combination with CP, the anthocyanin content was not further reduced. The firmness in the control group was 4.21 and 3.53 N on days 0 and 3, respectively. Similar to the results for TCD and anthocyanin content, firmness was not further reduced on day 0 after treatment with US-FC, US-PAA, US-FC + CP, or US-PAA + CP, and after storage for 3 days, a non-significant difference was observed between the four groups and the control.

### 3.5 Changes in reactive oxygen species (ROS) levels and antioxidant enzyme activity after different treatments

**Fig. 7:**
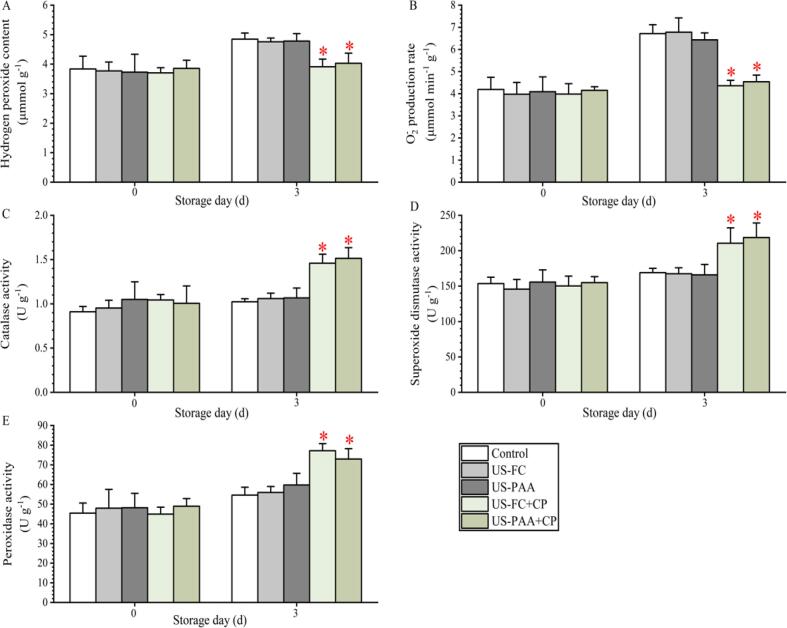
Effects of different combinations on reactive oxygen species metabolism and antioxidant enzyme activity in blueberries. (A) Hydrogen peroxide. (B) ${\text{O}}_{2}^{-}$ production rate. (C) Catalase. (D) Superoxide dismutase. (E) Peroxidase. The asterisk above the column indicates a significant difference (*P* < 0.05) with control in the same group. US, Ultrasound; PAA, peracetic acid; FC, free chlorine; CP, cold plasma.

As shown in Fig. 7A and 7B, the H_2_O_2_ content and the production rate of ${\text{O}}_{2}^{-}$ in the control group were 3.84 μM g^−1^ and 4.19 μM g^−1^ min^−1^, respectively. After treatment with four groups, the above two indicators did not significantly change on day 0. However, after storage for 3 days, the H_2_O_2_ content in US-FC + CP and US-PAA + CP groups were 3.91 and 4.03 μM g^−1^ and the production rate of ${\text{O}}_{2}^{-}$ was 4.36 and 4.54 μM g^−1^ min^−1^, respectively. These two indicators in US-FC + CP and US-PAA + CP group were significantly lower than those in the control, US-FC, and US-PAA groups, in contrast with the non-significant change observed on day 0. The CAT, SOD, and POD concentrations are shown in Fig. 7C, D, and E, respectively. Similar to the analysis of H_2_O_2_ and ${\text{O}}_{2}^{-}$ content, the activities of CAT, SOD, and POD in the control group were 0.91, 153.57, and 45.45 U g^−1^ on day 0, respectively, and no significant difference was observed between the control and treatment groups. On day 3, the activity of these three antioxidant enzymes in the combination groups was significantly higher than that in the control, US-FC, and US-PAA groups, in contrast with the ROS (i.e., H_2_O_2_ and ${\text{O}}_{2}^{-}$) content results on day 3.

## 4 Discussion

The shelf life of fresh products, especially, packaged ready-to-eat products, is short. For example, in the Lawson supermarket in China, ready-to-eat produce is recalled when the shelf life exceeds 24 h. As such, in most studies, the disinfection efficacy against foodborne pathogens on produce was determined only on day 0 [21], [32], [35], [36], [37], [38]. In this study, we analyzed changes in quality and microbial reduction on days 0 and 3.

After washing, fresh produce subsequently comes into contact with air, operators, conveyor belts, and spinner, which might reduce the disinfection effect of washing; thus, in-package disinfection methods are being explored in the field of food science. As a type of CP, a plasma jet is usually used to directly disinfect the fresh produce [39], [40], [41]; however, it is ineffective for packaged produce. Thus, plasma jet disinfection is consistent with the washing methods, which are all performed before packaging. In this study, DBD plasma was used for the in-package disinfection. During DBD processing, excited gas molecules were characterized, including ROS, such as ozone, atomic oxygen, superoxide anions, and reactive nitrogen species (RNS), such as excited nitrogen, atomic nitrogen, and nitric oxide [26], [41], [42]. To the best of our knowledge, few studies have used DBD plasma to disinfect produce under pulsed-wave control, which increases the incidence of package breakdown and causes quality loss. In this study, plasma was generated at different pulse frequencies, and 10 kHz frequency was selected as the control. Interestingly, we found that although the pulse frequency was much smaller than that of the control, the output power was similar or higher than that of the control, and the highest disinfection efficacy was observed in the range of 400–800 Hz, which was significantly higher than that of the control. This might be because even increasing the plasma discharge frequency did not cause further ROS and RNS production.

Processing time is an important factor in the minimal processing industry because it directly affects production efficiency. The treatment times of US-FC + CP and US-PAA + CP were consistent with those of US-FC and US-PAA (i.e., 2 min), and the disinfection efficacy was significantly improved, with a ∼ 2.0 log CFU/g reduction in for the two pathogens used in this study. However, in previous studies, the processing time of US + chemical agents was generally 5–10 min, with a microbial reduction ranging from 1 to 3 log CFU/g [8]. Moreover, it was found that pathogens cannot be completely inactivated in washing water when US is combined with certain agents (e.g., carbonated water, citral, Tween-20, and SDS) [10], [12], [21]. It has been demonstrated that FC at 10 ppm and PAA at 80 ppm can completely inactivate the pathogen in washing water during 1 min of washing [18], [19], and two concentrations were used in the present study.

**Fig. 8:**
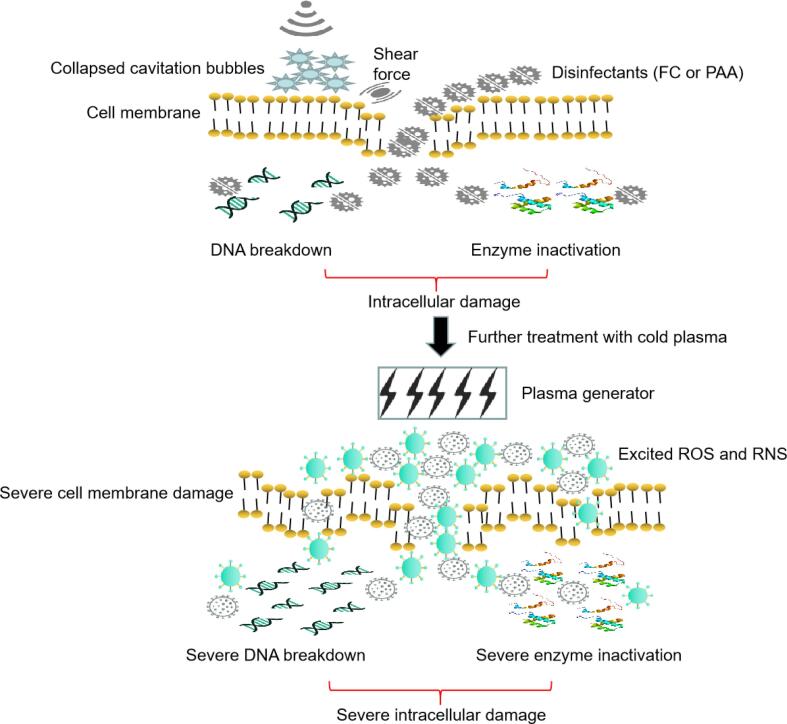
Potential mechanism underlying the antibacterial action of ultrasound-free chlorine combined with cold plasma and ultrasound-peracetic acid combined with cold plasma. FC: free chlorine; PAA: peracetic acid; ROS: reactive oxygen species; RNS: reactive nitrogen species.

US can induce the generation of cavitation bubbles, and the collapsing bubbles can generate shear force and instantaneous high pressure, which can lead to sonoporation of the bacterial membrane [7], [43]. The antibacterial effect of PAA and FC results in cell membrane damage via oxidation along with intracellular damage including enzyme inactivation and DNA damage [5], [33], [44]. Additionally, the disinfection efficacy of US-FC and US-PAA was improved compared to that of US, FC, and PAA. This improvement was associated with the acceleration of membrane and intracellular damage. Similarly, Zhao et al. [45] observed improved antibacterial activity against *E. coli* K12 and *Listeria monocytogenes* on mackerel fillets after treatment with US + PAA. The mechanism underlying the antibacterial effect of CP has not been determined. The main reasons for this are: (1) The excitation process is complex and the half-life of the resulting excited molecules is very short; correspondingly, the mechanism is complex and diverse; (2) if CP is used to disinfect bacteria present on the produce or contact surface, due to biofilm formation, the cells cannot be collected for further analysis. Instead, if CP is used to treat a bacterial suspension, the excitation process is different, and the plasma will induce aqueous solution excitation to generate ozone, nitrate, nitrite, and hydrogen peroxide [46], which is different from the effect of CP on gaseous materials. However, there are some popular hypotheses regarding the antibacterial effect of CP. Excited ROS and RNS accumulate on the cell membrane and impart an electrostatic force, which leads to lipid peroxidation of the cell membrane [47], [48]. At the same time, reactive species diffuse through the cell membrane and induce DNA oxidation and protein denaturation [41], [49]. CP can simultaneously cause cell membrane and intracellular damage, which is different from the effect of US-FC and US-PAA. Therefore, cell membrane damage was accelerated and intracellular components such as enzymes and DNA were damaged by US-induced shear force and the oxidative effects of FC and PAA (Fig. 8). The combination of US and FC/PAA with CP resulted in excited ROS and RNS, which disrupted the cell membrane further. This led to more severe cell membrane and intracellular damage, compared with that induced by US-FC and US-PAA, prompting greater reduction in microbial counts (Fig. 8).

Besides the decay caused by microbes, food quality is also an important factor affecting consumer choices. It has been reported that the low-frequency US can lead to loss of fresh produce quality. Santos et al. [50] processed fresh-cut mangoes for 30 min at 25 kHz and found that the color, firmness, soluble solids, and sugar content were significantly lower than those of the control mangoes on day 7. When the processing time was 1 min and the US frequency was 25 kHz, the visual and texture values of romaine lettuce were significantly lower than those of the control, whereas quality loss was not observed in iceberg lettuce [9]. Oxidizing sanitizers at low concentrations will not lead to quality loss [31]. In the present study, we found that US + 10 ppm FC and US + 80 ppm PAA did not lead to additional loss of firmness, TCD, and anthocyanin content, which is consistent with the results of Wang et al [30]. Excited ROS and RNS have a potential risk of degrading anthocyanins and lowering the firmness of blueberries. As reported by Hu et al. [26], a decrease in firmness and anthocyanin content was observed after treatment with CP for 20 min. The loss of anthocyanin after CP treatment was also observed by Sarangapani et al. [51], who employed a processing time of 5 min. In the present study, the anthocyanin content, TCD, and firmness were not altered after US-FC + CP and US-PAA + CP treatments, which was attributed to the short processing time and pulsed frequency control.

The accumulation of ROS (H_2_O_2_ and ${\text{O}}_{2}^{-}$) in fruits accelerates plant senescence and leads to loss of quality, such as softening and decay [34]. As living organisms, fruits are protected against oxidative stress by stimulating the antioxidant enzymes (CAT, SOD, and POD) to react with ROS. SOD is responsible for the breakdown of superoxide anions. The resulting H_2_O_2_ is then catalyzed by POD and CAT to generate water and oxygen [52]. Low frequency as a surface disinfection method may cause mechanical damage to fresh produce, and it has been reported that ROS production is accelerated and antioxidant enzyme expression is stimulated after mechanical damage [53]. Here, after treatment with US-FC and US-PAA for 2 min, ROS (H_2_O_2_ and ${\text{O}}_{2}^{-}$) production and antioxidant enzyme activity (CAT, POD, and SOD) were consistent with those of the control, indicating that mechanical damage did not occur. To prolong shelf life, the abiotic stress method was previously used, where stimulation of antioxidant enzymes using oxidizing agents, such as ozone, was successfully applied to papaya, kiwifruit, raspberry, and orange [54], [55], [56], [57]. In the present study, after combining the treatments with CP, we observed a decrease in ROS levels and an increase in antioxidant enzyme activity on day 3, which was associated with the excited ROS and RNS, stimulating the antioxidant signaling in blueberries.

## 5 Conclusion

In this study, we combined US-assisted washing with in-package disinfection, and explored the disinfection efficacy and quality changes of blueberries. The main findings are as follows:

(1) When US was combined with PAA and FC, the disinfection efficacy improved and was significantly higher than that of US, PAA, and FC alone.
(2) The best disinfection efficacy of CP was observed at a pulse frequency of 400–800 Hz, and microbial reduction was positively correlated with plasma output power in the range of 50–800 Hz.
(3) For the same processing time, the disinfection efficacy of US-FC + CP and US-PAA + CP treatments against *E. coli* O157:H7, *S.* Typhimurium, AMC, and M&Y was significantly higher than that of US-FC and US-PAA treatments.
(4) US-FC + CP, US-PAA + CP, US-FC, and US-PAA treatment did not lead to quality loss.
(5) Compared with US-FC and US-PAA, combination treatment with CP lowered the ROS content by stimulating antioxidant enzymes.

The underlying mechanism of the antibacterial effect of CP has not been revealed; therefore, that of US-FC + CP and US-PAA + CP against pathogens could not be determined. However, with the development of sequencing technologies, the metatranscriptome method has gradually matured. In future studies, the underlying mechanism of the antibacterial effect of these two combinations can be explored from the perspective of changes in the transcriptome of naturally present microbes on fresh produce.

### CRediT authorship contribution statement

**Jiayi Wang:** Conceptualization, Supervision, Funding acquisition, Writing – original draft, Writing – review & editing. **Zhaoxia Wu:** Data curation, Writing – review & editing.

## Declaration of Competing Interest

The authors declare that they have no known competing financial interests or personal relationships that could have appeared to influence the work reported in this paper.
